# 2-Supernilpotent Mal’cev algebras

**DOI:** 10.1007/s00605-013-0541-y

**Published:** 2013-08-31

**Authors:** Nebojša Mudrinski

**Affiliations:** 1 Institut für Algebra, Johannes Kepler Universität, 4040 Linz, Austria; 2 Department of Mathematics and Informatics, Faculty of Sciences, University of Novi Sad, Trg Dositeja Obradovića 4, 21000 Novi Sad, Serbia

**Keywords:** Commutators, Nilpotent, Mal’cev algebra, Expanded group, 08A05

## Abstract

In this note we prove that a Mal’cev algebra is 2-supernilpotent ([1, 1, 1] =  0) if and only if it is polynomially equivalent to a special expanded group. This generalizes Gumm’s result that a Mal’cev algebra is abelian if and only if it is polynomially equivalent to a module over a ring.

## Introduction

It is well known that the commutator $[a,b]=a^{-1}b^{-1}ab$ of two elements $a$ and $b$ of a group $\mathbf G$ can be seen as a “measure” how far are $a$ and $b$ from commuting according to the group operation of $\mathbf G$. Thus, the normal subgroup $[G,G]$ generated by all such commutators “measures” how far is the group $\mathbf G$ from an abelian group. Namely, $[G,G]=0$ if and only if $\mathbf G$ is abelian. The concept of commutators of normal subgroups has been generalized to a binary operation of the congruence lattice of arbitrary algebras in congruence modular varieties by Smith, Freese, McKenzie, Hagemann, Herrmann,...One can find more details in [5]. A special case of congruence modular varieties are congruence permutable varieties. In congruence permutable varieties each algebra has a Mal’cev term. A *Mal’cev term* of an algebra $\mathbf A$ is a ternary term operation $d$ of $\mathbf A$ that satisfies $d(x,y,y)=d(y,y,x)=x$ for all $x,y\in A$. An algebra with a Mal’cev term we call a *Mal’cev algebra*. What $[1,1]=0$ in Mal’cev algebras means has been answered by Gumm, Hagemann and Herrmann, see [4, Theorem 13.4]. They proved that a Mal’cev algebra is *abelian* $([1,1]=0)$ if and only if it is polynomially equivalent to a module over a ring. Here, we call two algebras $\mathbf A$ and $\mathbf B$ on the same domain *polynomially equivalent* if they have the same set of polynomial operations. In 2001, A. Bulatov has generalized the binary commutator operations to $n$-ary commutator operations $[\bullet ,\bullet ,\ldots ,\bullet ]$ on congruence lattices of Mal’cev algebras, for each $n\in \mathbb N$, see [3]. We will restrict ourselves to the ternary commutators. The aim of this note is to answer what $[1,1,1]=0$ means in Mal’cev algebras. We want to characterize all Mal’cev algebras with such a property, because it could be expected that $[1,1,1]=0$ implies that the algebra is polynomially equivalent to a well studied structure. According to the property (HC3) in [2] we know that $[1,1,1]\le [1,1]$. Therefore, we have that each abelian Mal’cev algebra satisfies the condition $[1,1,1]=0$. In Theorem 3.3 we prove that every Mal’cev algebra with $[1,1,1]=0$ is an expanded group.

In accordance with [2, Definition 7.1], Mal’cev algebras that satisfy $[1,1,1]=0$ are called *2-supernilpotent*. As it has been defined in [5] algebras that satisfy $[1,[1,1]]=0$ are called *2-nilpotent*. In expanded groups we deal with ideals rather then with congruences, because the corresponding commutator operations act in the same way according to [2, Corollary 6.12]. An expanded group $\mathbf V$ is 2-nilpotent if $[V,[V,V]]=0$ and 2-supernilpotent if $[V,V,V]=0$. Let us just mention that all 2-nilpotent algebras are nilpotent by definition, see [5].

## Special expanded groups

A *polynomial* of an algebra $\mathbf A$ is an operation obtained by composition of projections, fundamental and constant operations of $\mathbf A$, see [6, Definition 4.4]. The set of all $n$-ary polynomials of an algebra $\mathbf A$ we denote by $\mathsf{Pol}_n\mathbf A$ for all $n\in \mathbb N$.

### **Definition 2.1**

 Let $\mathbf V =(V,+,-,0,F)$ be an expanded group and let $n\in \mathbb N$. We call an $n$-ary polynomial $f$ of $\mathbf V$ *absorbing* if $f(a_{1},\ldots ,a_{n})=0$ whenever there exists an $i\in \{1,\ldots ,n\}$ such that $a_i=0$. The set of unary absorbing polynomials of $\mathbf V$ we denote by $P_0(\mathbf V )$ and the set of binary absorbing polynomials of $\mathbf V$ we denote by $CP(\mathbf V )$.

More generally, for an algebra $\mathbf A$ and an $n\in \mathbb N$, $(a_{1},\ldots ,a_{n})\in A^n$, $a\in A$ we say that an $n$-ary polynomial $p$ is *absorbing at* $(a_{1},\ldots ,a_{n})$ *with value* $a$ if $p(x_{1},\ldots ,x_{n})=a$ whenever there exists an $i\in \{1,\ldots ,n\}$ such that $x_i=a_i$. 

### **Lemma 2.2**

 Let $\mathbf V$ be an expanded group with a group reduct $(V,+,-,0)$, and let $n\in \mathbb N$. For $i\le n$ let $\pi _i^n:V^n\rightarrow V$ be the $i$-th projection. Then the group $(\mathsf{Pol}_n\mathbf{V },+,-,0)$ is generated by constants and

$$\begin{aligned}&\bigcup _{k=1}^n\{f(\pi _{i_1}^n,\ldots ,\pi _{i_k}^n):f \text { is a nonzero absorbing polynomial of }\mathbf V \text { of degree } k,\\&\qquad i_1,\ldots ,i_k\in \{1,\ldots ,n\} \}. \end{aligned}$$

### *Proof*

 See [1, Lemma 3.1]. In [1, p. 260] each $n$-ary commutator polynomial is exactly a polynomial $f(\pi _{i_1}^n,\ldots ,\pi _{i_k}^n)$ where $k\le n$ and $f$ is a nonzero $k$-ary absorbing polynomial defined in Definition 2.1 or constant. $\square$

### **Lemma 2.3**

 Let $\mathbf V =(V,+,-,0,F)$ be an expanded group such that

1. $F$ is the set of at most binary absorbing operations on $V$,
2. Every absorbing operation in $\mathsf{Pol}_2(\mathbf V )$ is distributive with respect to $+$, and
3. $$\mathbf V $$ is $$2$$-nilpotent.

Then $\mathbf V$ is $2$-supernilpotent. 

### *Proof*

First, we show that for all $k\in \mathbb N$ and $f\in \mathsf{Pol}_k(\mathbf V )$ there exist $c\in V$, $p_i\in P_0(\mathbf V )$ and $q_{ij}\in CP(\mathbf V )$ for $1\le i<j\le k$ such that

2.1 $$\begin{aligned} f(x_{1},\ldots ,x_{k})=c+p_1(x_1)+\cdots +p_k(x_k)+\sum _{1\le i<j\le k}q_{ij}(x_i,x_j). \end{aligned}$$

Let $P_k$ be the set of operations as in (2.1). Obviously $P_k\subseteq \mathsf{Pol}_k(\mathbf V )$. To prove $\mathsf{Pol}_k(\mathbf V )\subseteq P_k$, we need to show that the set $P_k$ contains projections and constants and that is closed under the basic operations of $\mathbf V$. Projections are in $P_0(\mathbf V )$ and therefore in $P_k$. Constants are in $P_k$ because $P_0(\mathbf V )$ and $CP(\mathbf V )$ contain zero polynomials. Clearly, $P_k$ is closed under $+$ and $-$ since $[c,p(x_i)]=r(x_i)$, for some $r\in P_0(\mathbf V )$, $[c,q_{ij}(x_i,x_j)]=s(x_i,x_j)$ for some $s\in CP(\mathbf{V })$ and $[p(x_i),p(x_j)]=t(x_i,x_j)$ for some $t\in CP(\mathbf{V })$. Furthermore, $[q_{ij}(x_i,x_j),p_l(x_l)]$ and $[q_{ij}(x_i,x_j),q_{lt}(x_l,x_t)]$ are zero polynomials because $\mathbf V$ is $2$-nilpotent. Therefore, in the sum of two operations in the form of (2.1) one can permute all summands to obtain again the form as in (2.1), because $P_0(\mathbf V )$ and $CP(\mathbf V )$ are closed under $+$ and $-$. To show that $P_k$ is closed under all $q\in CP(\mathbf{V })$ we note that for all $q\in CP(\mathbf{V })$ and $f,f'\in P_k$ we have $q(f,f')\in P_k$ by the distributivity of $q$ and the $2$-nilpotence of $\mathbf V$.

It remains to show that $P_k$ is closed under any unary operation $g\in F$. Note that $g'(x,y):=g(x+y)-g(x)-g(y)$ is in $CP(\mathbf V )$, hence distributive. By induction it follows that for every $n$ there exist $c_{ij}\in CP(\mathbf V )$ such that

2.2 $$\begin{aligned} g(x_1+\cdots +x_n)=g(x_1)+\cdots +g(x_n)+\sum _{1\le i<j\le n}c_{ij}(x_i,x_j). \end{aligned}$$

For $f$ as in (2.1) this yields

2.3 $$\begin{aligned} gf(x_1,\ldots ,x_k)&= g(c)+gp_1(x_1)+\cdots +gp_k(x_k)+\sum _{1\le i<j\le k}gq_{ij}(x_i,x_j)\nonumber \\&+\sum _{1\le i\le k}c_i(c,p_i(x_i))+\sum _{1\le i<j\le k}c_{ij}(p(x_i),p(x_j)) \end{aligned}$$

for some $c_i,c_{ij}\in CP(\mathbf V )$. Note that the other terms vanish by $[V,[V,V]]=0$. Thus $gf\in P_k$. We have proved $P_k=\mathsf{Pol}_k(\mathbf V )$.

Now we know that for every ternary polynomial operation $p$ on $\mathbf V$ there exist a $c\in V$, $f,g,h\in P_0(\mathbf V )$ and $r,s,t\in CP(\mathbf{V })$ such that

2.4 $$\begin{aligned} p(x,y,z)=c+f(x)+g(y)+h(z)+r(x,y)+s(x,z)+t(y,z) \end{aligned}$$

for all $x,y,z\in V$. Using the absorbing property of $p,f,g,h,r,s$ and $t$ we obtain the following. First, $c=0$ because $p(0,0,0)=0$ from (2.4). Then, we substitute $y=z=0$ in (2.4) and obtain $f(x)=0$. Analogously, we have $g(y)=h(z)=0$. It remains $p(x,y,z)=r(x,y)+s(x,z)+t(y,z)$. We have $t(y,z)=0$, because $0=p(0,y,z)$. Analogously, we obtain $r(x,y)=s(x,z)=0$. Hence, every ternary absorbing polynomial operation is constant 0. Whence, $\mathbf V$ is $2$-supernilpotent, by [2, Corollary 6.12]. $\square$

## Mal’cev algebras

### **Proposition 3.1**

 (cf. [5, Corollary 7.4]) Let $\mathbf A$ be a nilpotent Mal’cev algebra with a Mal’cev term $d$ and let $o\in A$. Then for all $a_1,a_2,b_1,b_2\in A$ there exist $x,y\in A$ such that $d(x,o,a_1)=b_1$ and $d(a_2,o,y)=b_2$. 

### *Proof*

The function $x\mapsto d(x,o,a_1)$ is bijective for all $a_1\in A$, by [5, Corollary 7.4]. Hence, the equation $d(x,o,a_1)=b_1$ has a unique solution for all $a_1,b_1\in A$. We know that $D(x,y,z):=d(z,y,x)$ for all $x,y,z\in A$ is also a Mal’cev term of $\mathbf A$. Therefore, $y\mapsto D(y,o,a_2)$ is bijective for all $a_2\in A$ by [5, Corollary 7.4]. Hence, $y\mapsto d(a_2,o,y)$ is bijective for all $a_2\in A$. Whence, the equation $d(a_2,o,y)=b_2$ has a unique solution for all $a_2,b_2\in A$. $\square$

### **Lemma 3.2**

 (cf. [7, Theorem 1.2]) Every semigroup $(G,+)$ such that the equations $a_1+x=b_1$ and $y+a_2=b_2$ are solvable for all $a_1,a_2,b_1,b_2\in A$, is a group. 

### *Proof*

 See [7, Definition 1.1, Theorem 1.2]. $\square$

### **Theorem 3.3**

 For a Mal’cev algebra $\mathbf A$ the following are equivalent:

1. $$\mathbf A $$ is $$2$$-supernilpotent $$([1,1,1]=0)$$
2. $\mathbf A$ is polynomially equivalent to an expanded group $\mathbf V =(A,+,-,0,F)$ such that
  - $F$ is a set of at most binary absorbing operations on $\mathbf V$,
  - Every absorbing operation in $\mathsf{Pol}_2(\mathbf V )$ is distributive with respect to $+$ on both arguments, and
  - $$\mathbf V $$ is $$2$$-nilpotent.

### *Proof*

$(1)\Rightarrow (2)$ Using [2, (HC8)] we obtain $[1,[1,1]]=0$. Therefore, $\mathbf A$ is a 2-nilpotent Mal’cev algebra. Let $o\in A$ and let $d$ be a Mal’cev term. We define $+:A^2\rightarrow A$ by

$$\begin{aligned} x+y:=d(x,o,y) \end{aligned}$$

for all $x,y\in A$. From Proposition 3.1 we know that the equations $a_1+x=b_1$ and $y+a_2=b_2$ are solvable for all $a_1,a_2,b_1,b_2\in A$. Let us show that $+$ is associative. We observe that the polynomial

$$\begin{aligned} p(x,y,z):=d(d(d(x,o,y),o,z),d(x,o,d(y,o,z)),o) \end{aligned}$$

for all $x,y,z\in A$ is an absorbing polynomial at $(o,o,o)$ with value $o$. Therefore, $(p(a,b,c),o)\in [1,1,1]$ for all $a,b,c\in A$ by [2, Lemma 6.9]. Using the assumption $[1,1,1]=0$, we obtain $p(a,b,c)=o$. Equivalently,

$$\begin{aligned} d(d(d(a,o,b),o,c),d(a,o,d(b,o,c)),o)=o. \end{aligned}$$

By [5, Corollary 7.4] we have that $x\mapsto d(x,d(a,o,d(b,o,c)),o)$ is a bijective mapping of $A$. Hence, $d(d(a,o,b),o,c)=d(a,o,d(b,o,c))$, because $d(d(d(a,o,b),o,c),d(a,o,d(b,o,c)),o)=d(d(a,o,d(b,o,c)),d(a,o,d(b,o,c)),o)$. Using the recently introduced notation we have proved $(a+b)+c=a+(b+c)$. Now using Lemma 3.2 we obtain that $+$ is a group operation with the neutral element $o$. We denote the inverse of an element $a\in A$ by $-a$. This is a polynomial operation given by

$$\begin{aligned} -x:=d(o,d(d(o,x,o),o,x),d(o,x,o)) \end{aligned}$$

for all $x\in A$, by [5, Lemma 7.3]. We have proved that $\mathbf A$ is polynomially equivalent to an expanded group $\mathbf V$ with the group reduct $(A,+,-,o)$. Now, we shall prove that $\mathbf A$ is polynomially equivalent to the expanded group $\mathbf{V }:=(A,+,-,o,F)$, where $F:=P_o(\mathbf{A })\cup CP(\mathbf{A })$. We have already that all fundamental operations of $\mathbf V$ are polynomials of $\mathbf A$.

By [2, (HC3)], we know that $[\,\underbrace{1,\ldots ,1}_n\,]=0$ for all $n\ge 3$. Hence, $f=0$ for all $n$-ary absorbing polynomial operations $f$ of $\mathbf A$ if $n\ge 3$, by [2, Corollary 6.12]. Hence, the set of all non-constant absorbing polynomial operations of $\mathbf A$ is $F:=P_o(\mathbf{A })\cup CP(\mathbf{A })$. Using Lemma 2.2 we obtain that each polynomial of $\mathbf A$ is also a polynomial of $\mathbf V$.

Let $f\in CP(\mathbf{V })$. One can easily see that the polynomial $q$ defined by

$$\begin{aligned} q(x,y,z):=f(x,y+z)-f(x,z)-f(x,y) \end{aligned}$$

for all $x,y,z\in A$ is absorbing at $(o,o,o)$ with value $o$. Therefore, $(q(a,b,c),o)\in [1,1,1]$ by [2, Corollary 6.9] for all $a,b,c\in A$. Hence, $q(a,b,c)=o$ or equivalently,

$$\begin{aligned} f(a,b+c)=f(a,b)+f(a,c) \end{aligned}$$

for all $a,b,c\in A$, by the assumption $[1,1,1]=0$. The proof for the distributivity on the first argument is analogous.

$(2)\Rightarrow (1)$ Polynomially equivalent algebras have the same congruence lattice and the same commutator operations acting on the congruence lattice, by [2, Corollary 6.11]. Therefore, $\mathbf A$ is $2$-supernilpotent by Lemma 2.3. $\square$
